# Production and Partial Characterization of *α*-Amylase Enzyme from* Bacillus* sp. BCC 01-50 and Potential Applications

**DOI:** 10.1155/2017/9173040

**Published:** 2017-01-12

**Authors:** Altaf Ahmed Simair, Abdul Sattar Qureshi, Imrana Khushk, Chaudhry Haider Ali, Safia Lashari, Muhammad Aqeel Bhutto, Ghulam Sughra Mangrio, Changrui Lu

**Affiliations:** ^1^College of Chemistry, Chemical Engineering and Biotechnology, Donghua University, Shanghai 201620, China; ^2^Institute of Biotechnology and Genetic Engineering, University of Sindh, Jamshoro 76080, Pakistan; ^3^Department of Chemical Engineering, University of Engineering & Technology, KSK Campus, Lahore 54890, Pakistan; ^4^State Key Laboratory of Bioreactor Engineering and Institute of Applied Chemistry, East China University of Science and Technology, Shanghai 200237, China

## Abstract

Amylase is an industrially important enzyme and applied in many industrial processes such as saccharification of starchy materials, food, pharmaceutical, detergent, and textile industries. This research work deals with the optimization of fermentation conditions for *α*-amylase production from thermophilic bacterial strain* Bacillus* sp.* BCC 01-50* and characterization of crude amylase. The time profile of bacterial growth and amylase production was investigated in synthetic medium and maximum enzyme titer was observed after 60 h. In addition, effects of different carbon sources were tested as a substrate for amylase production and molasses was found to be the best. Various organic and inorganic compounds, potassium nitrate, ammonium chloride, sodium nitrate, urea, yeast extract, tryptone, beef extract, and peptone, were used and beef extract was found to be the best among the nitrogen sources used. Temperature, pH, agitation speed, and size of inoculum were also optimized. Highest enzyme activity was obtained when the strain was cultured in molasses medium for 60 h in shaking incubator (150 rpm) at 50°C and pH 8. Crude amylase showed maximal activity at pH 9 and 65°C. Enzyme remained stable in alkaline pH range 9-10 and 60–70°C. Crude amylase showed great potential for its application in detergent industry and saccharification of starchy materials.

## 1. Introduction

Amylase hydrolyzes the starch molecules in dextrin, maltose, and finally glucose units. The enzymes are of industrial importance due to their commercial applications in starch liquefaction, paper, desizing of textile fabrics, preparing starch coatings of paints, removing wallpaper, brewing industry, sugar induction by the production of sugar syrups, pharmaceuticals, and preparing cold water dispersible laundry. They can be produced by micro- and macroorganisms [1]. Microbial amylase is preferred over other kinds of amylase obtained from plants and animals due to its biochemical versatility, higher production rate, stability, and easy availability of huge number of microbial strains. Currently, amylase production has reached up to 65% of enzyme market in the world and is continuously increasing [2].

Recently, starch saccharification, the major application of amylase, has completely replaced chemical utilization with amylase enzyme hydrolysis. Saccharification is performed at elevated temperature and thermophilic microorganisms could be most promising candidates for amylase production because these strains will produce thermostable amylase. This is why, still, search for novel microbial strains is continued to fulfill industrial demands of enzymes. In addition, amylase is supplemented in local detergents due to high alkaline pH stability required for industries [3]. Moreover, thermophilic amylase is required for other applications in the production of sweeteners from starch and saccharification of starch for biochemical production [4].

*Bacillus* genus is well known and famous for alpha-amylase production and several* Bacillus* strains such as* B. stearothermophilus*,* B. subtilis*,* B. cereus*,* B. licheniformis*, and* B. amyloliquefaciens* are isolated and screened for amylase production [5]. Some* Bacillus* strains are involved in raw starch degradations [6–9]. Still, many are being searched to obtain the highest production with unique industrial properties. Enzyme production could be improved through optimizing culture conditions and with the latest techniques including induced mutation and gene cloning. Amylase production was carried out by using various agroindustrial residues including rice husk, wheat straw, and rice straw as cost-effective carbon sources. Utilization of these waste materials provide a low-cost medium for amylase production and solve the environmental pollution problem. Previous studies have applied agroindustrial residues such as molasses, date syrup, and fruit waste for production of several economically important enzymes [10–16]. The main objective of this study was to evaluate new thermophilic* Bacillus* sp. BCC 01-50 for extracellular amylase production using molasses as a cost-effective carbon source. Crude amylase applicability showed great potential for its application in detergent industry and saccharification of starchy materials. Results obtained herein are more promising for the inexpensive production of amylase by using agroindustrial residue as cost-effective carbon source for biotechnological applications.

## 2. Materials and Methods

### 2.1. Microorganism and Cultivation Conditions

Bacterial strains previously isolated, identified, and stored in our laboratory were screened for amylase production in starch agar medium. Medium contained soluble starch 20 g/L, peptone 20 g/L, and agar 20 g/L, then culture was spread, and plates were incubated for 48 h. After 48 h, incubation plates were flooded with Gram's iodine solution to observe clear zone that was indication of starch hydrolysis. Strain* Bacillus* sp.* BCC-01-50* was selected on the basis of the large clear zone for further study to evaluate its fermentability in agroindustrial residue and characterization of the crude enzyme for its biotechnological applications. The strain was incubated in LB medium containing 10 g/L of tryptone, 5 g/L of yeast extract, and 10 g/L of sodium chloride for 24 h at 37°C in shaking incubator. After 24 h, 10 (% v/v) culture was inoculated in the fermentation medium (glucose 20 g/L, yeast extract 10 g/L, MgSO_4_·7H_2_O 1.0 g/L, and KH_2_PO_4_ 2 g/L), and culture was incubated at 37°C for 120 h in shaking incubator. Samples were collected at a regular interval of 12 h, and growth (OD) was measured by a spectrophotometer. The culture broth was centrifuged at 12,100*g* for 10 min to collect supernatant and was used for biochemical analysis of *α*-amylase activity, total protein, and total sugars. All media were sterilized at 121°C, 15 lb/inch^2^ pressure for 20 minutes prior to inoculation.

### 2.2. Effect of Carbon Source

Effect of various carbon sources was checked by using glucose, date syrup, molasses, fructose, starch, lactose, galactose, and sucrose with w/v concentration (20 g/L) for microbial growth and enzyme production. 250 mL Erlenmeyer Flasks with 50 mL fermentation medium were inoculated with 10 (% v/v) seed culture and inoculated at 37°C for 60 h. In another set of experiment, different concentrations of molasses were also tested. At the end of fermentation, culture broth was collected by centrifugation (12,100*g* for 10 min at 10°C) and the broth was used for biochemical analysis.

### 2.3. Effect of Nitrogen Source

After optimizing carbon sources, various organic and inorganic compounds were evaluated as a nitrogen source for cell growth and amylase production. Potassium nitrate, ammonium chloride, sodium nitrate, urea, yeast extract, tryptone, beef extract, and peptone were supplemented in synthetic medium with 10 g/L (w/v) concentration. Amylase titer was determined as described above. In a separate set of experiment, the effect of beef extract concentrations on amylase yield and bacterial growth was determined.

### 2.4. Influence of Initial pH and Temperature

Amylase activity was checked at different initial pH values ranges 5.0–11.0 and temperature range 30–60°C. 250 mL flask containing 50 mL of enzyme production medium was inoculated with 10 (% v/v) inoculum from 24 h old seeds culture and was incubated in shaking incubator at 37°C for 60 h, while, in case of temperature optimization, pH was adjusted to 8.0 and other conditions remained unchanged. Amylase activity was determined from culture broth obtained after centrifugation (12,100*g* for 10 min at 10°C).

### 2.5. Size of Inoculum

To investigate the effect of inoculum size on cell growth and amylase activity, 50 mL of fermentation medium was inoculated with 24 h old mature seeds culture with v/v % (0.5, 1.0, 2.0, 4.0, 6.0, 8.0, and 10.0) and 8.0 pH was adjusted. After incorporation of seed culture, flasks were incubated in shaking incubator for 60 h at 50°C. After 60 h incubation, culture filtrate was collected and amylase activity was tested.

### 2.6. Characterization of Crude Amylase

Optimum pH requirement is very important for an enzyme-substrate reaction, so broader pH range 5.0–12.0 was investigated. The pH values were obtained with the addition to different buffer solutions: sodium acetate buffer, pH 5.0; phosphate buffer, pH 6.0–8.0; and glycine–NaOH buffer, pH 9.0–12.0. The reaction mixture was incubated at optimum temperature and activity was determined by DNS method. pH stability was determined by incubating enzyme in the above-mentioned buffers at 65°C for 1 h and, in terms of temperature and temperature stability after incubating enzyme at a different temperature range (30–95°C) for 1 h, residual amylase activity was measured under assay conditions.

### 2.7. Amylase Applicability

Amylase plays vital role in starch processing and in local detergent industries. Applicability of bacterial amylase was checked by determination of released glucose from starch hydrolysis during cloth washing. Experimental design was used as proposed by Shukla and Singh [17] and same design was applied in our previous research [18]. A piece of cotton cloth was stained with 0.5 mL starch solution (0.5%) and dried at 50°C for 30 min. The dried stained piece of cloth was dipped in a mixture of 0.5 mL crude enzyme and 5 mL of tap water. A piece of cloth in enzyme solution was incubated in a shaker (100 rpm) at 40°C for 20 min. After incubation, the cloth was squeezed inside the beaker and was incubated for 5 days. In comparison to other studies, our study requires less time and the more important crude enzyme is as effective as purified *α*-amylase. We observed that crude amylase is efficient for starch hydrolysis in a short time and suitable for local detergent formulations and as starch degrading enzyme.

The washout was used to estimate the reducing sugar with modified Anthrone's method reported by Shukla and Singh [17]. Same method was applied in another set of experiments with combination of 0.5% commercially available detergents (Ariel, Omo, Bright, Surf Excel, Bonus, and Rin) by replacing partially purified enzyme and with detergent + enzyme.

The starch amount was monitored by multiplying the glucose amount with 0.9 as reported by Shukla and Singh [17]. Washing efficiency can be expressed as

(1) $$\begin{array}{c} \%\text{ efficiency}=\frac{100\times A\times 0.9}{B}, \end{array}$$

where *A* is amount of glucose released after reaction (g/mL) in a washout and *B* is amount of starch (*μ*g/mL) applied for staining of clean cotton cloth piece.

Starch hydrolysis % was measured using the following equation proposed by Liu and Xu, and Hasjim et al. [19, 20]:

(2) $$\begin{array}{c} \text{Rh}\%=\left(\;\frac{A1}{A0}\;\right)\times 100, \end{array}$$

where *A*1 was the concentration of reducing sugars in clear solution (supernatant) after enzymatic hydrolysis reaction and *A*0 was the concentration of raw starch used in the reaction.

### 2.8. Amylase Assay

1.0 mL of sample and an equal amount of substrate (1.0% w/v soluble starch) were mixed thoroughly and test tubes were incubated at 37°C for 15 min in water bath. After 10 min, reaction was stopped by addition of 2.0 mL of DNS reagent and tubes were kept in boiling water bath for 5 min. Tubes were cooled at room temperature and absorbance was measured at 540 nm against substrate and enzyme blank. The blanks were prepared by replacing sample and substrate with the same amount of water. All other reagents were added in the same concentration.

### 2.9. Statistical Analysis

Data were expressed as means ± standard deviation (SD) for triple determinations. The differences among means were assessed via least significant difference (LSD) tests. The significance level was set at *p* < 0.05.

## 3. Results and Discussion

### 3.1. Optimization of Fermentation Conditions

**Figure 1:**
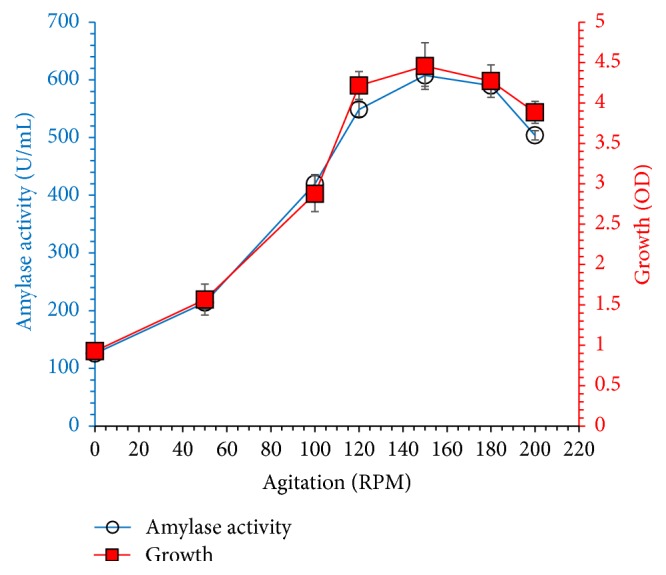
Effect of agitation speed (0–200 rpm) on amylase production and cell growth at 37°C, initial pH 7.0 for 48 h. The experiments were performed in triplicate and data presented in figure is average of three parallel experiments. Error bars are shown for standard deviation.

Figure 1 shows the effect of agitation speed on cell growth and amylase production. Data revealed that shaking is very essential for luxurious growth and higher enzyme titer. Both enzyme production and cell growth increased with agitation speed up to 150 rpm and then declined; it might be due to the damage of bacterial cells at higher shaking speed.

**Figure 2:**
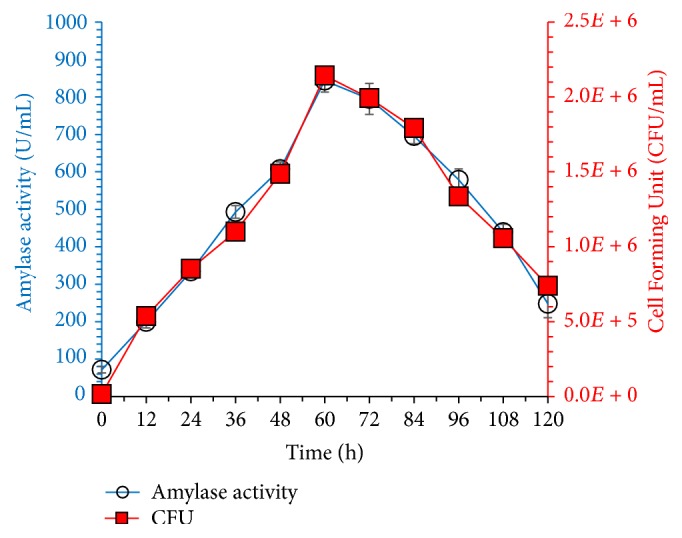
Batch profile of amylase production and* Bacillus* sp. BCC 01-50 growth in synthetic medium containing (g/L) glucose 20, peptone 10, MgSO_4_·7H_2_O 2, and KH_2_PO_4_ 3 incubated in shaking incubator at 37°C with initial pH 7.0 at 150 rpm agitation speed. The experiments were performed in triplicate and data presented in figure is average of three parallel experiments. Error bars are shown for standard deviation.

In our previous studies, molasses, a brown liquid obtained from sugar industry, has been applied for alkaline phosphatase [11–13] and protease production. Molasses is a nutrient rich component used as cost-effective carbon, nitrogen, and mineral source for growth regulation and biochemical production. Utilization of agroindustrial waste as substrate not only reduces the cost of enzyme production but also helps to reduce pollution problems occurring due to the accumulation of waste material. Effects of incubation time on the synthesis of amylase by* Bacillus* sp.* BCC 01-50* and cell forming unit (CFU/mL) are shown in Figure 2. Cell growth and enzyme concentration increased with time of incubation and reached to maximum after 60 h (843 U/mL); then growth and enzyme titer gradually decreased.  Table 1 shows the growth kinetics in terms of biomass production and glucose consumption in the synthetic medium. First part of study deals with optimization of fermentation conditions. Time of incubation results revealed that, on elongated incubation period, amylase concentration was reduced most probably due to the reduction in nutrients, accumulation of waste product, cell death, and catabolite repression [21]. Generally, a higher concentration of primary metabolites is achieved in exponential phase due to the availability of enough nutrients and important metabolites. Previously, highest amylase concentration was achieved after 72 h incubation; when wheat bran was used as substrate,* Aspergillus niger* BTM-26 and* Bacillus amyloliquefaciens* released maximum enzyme concentration after 48 h in submerged fermentation [2, 21].

**Figure 3:**
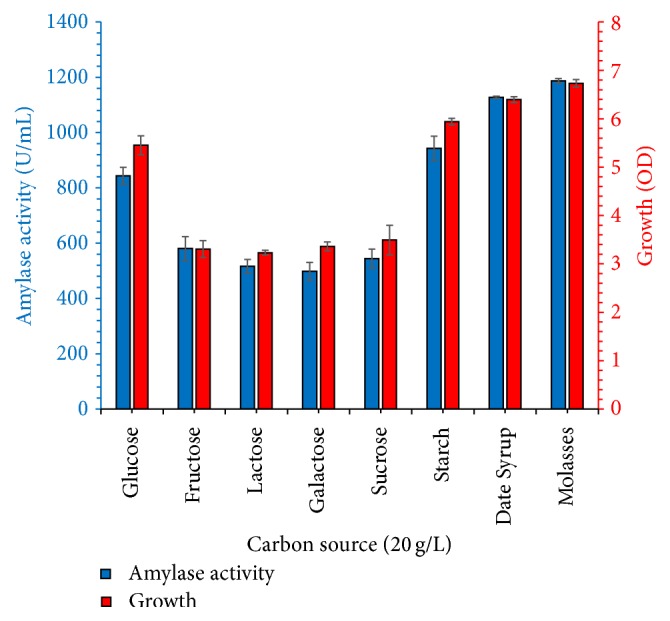
Effect of the carbon source (20 g/L initial concentration) on *α*-amylase production and bacterial growth at 37°C, initial pH of 7.0 for 60 h. The experiments were performed in triplicate and data presented in figure is average of three parallel experiments. Error bars are shown for standard deviation.

**Figure 4:**
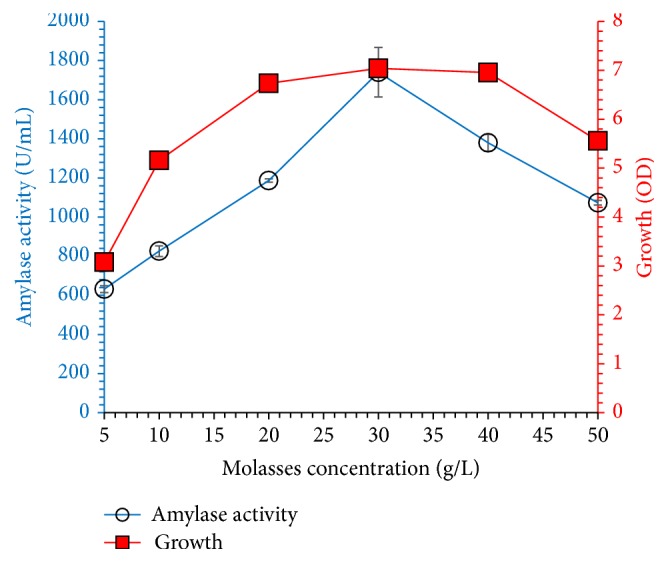
Effect of molasses concentration (5–50 g/L) on amylase yield titer and* Bacillus* sp. BCC 01-50 growth when incubated in shaking incubator at 37°C, initial pH of 7.0 for 60 h. The experiments were performed in triplicate and data presented in figure is average of three parallel experiments. Error bars are shown for standard deviation.

The effects of different carbon sources (molasses, date syrup, glucose, sucrose, fructose, starch, lactose and galactose) were investigated on growth and enzyme production results are shown in Figure 3. Bacterial growth and amylase yield were highest, when 20 g/L of molasses was used as a carbon source as compared to other carbon sources. This occurred probably due to the presence of growth promoters and other nutrients in molasses. In our study, molasses was found to be the best carbon source for microbial growth and amylase fermentation. This occurred probably due to the presence of growth promoters and other nutrients in molasses. Many researchers have produced *α*-amylase from too expensive pure sugars, but agroindustrial residues could be attractive carbon sources for the production of enzymes and other valuable products to reduce the cost of fermentation medium because more than 60% of cost belongs to carbon sources. So in present research study, for agroindustrial residue, molasses was utilized as a cost-effective carbon source and molasses medium secreted highest *α*-amylase yield. Abdullah et al. [2] have utilized different agroindustrial residues as a carbon source (coconut oil cake, rice bran, vegetable waste, banana peel, and wheat bran) and obtained maximum amylase concentration from wheat bran compared to other waste materials. In another set of experiment, the effect of molasses concentration (5–50 g/L) was tested. Amylase concentration increased with molasses up to 30 g/L; then a further increase of molasses lowered the yield probably due to carbon catabolic repression; results are shown in Figure 4.

**Figure 5:**
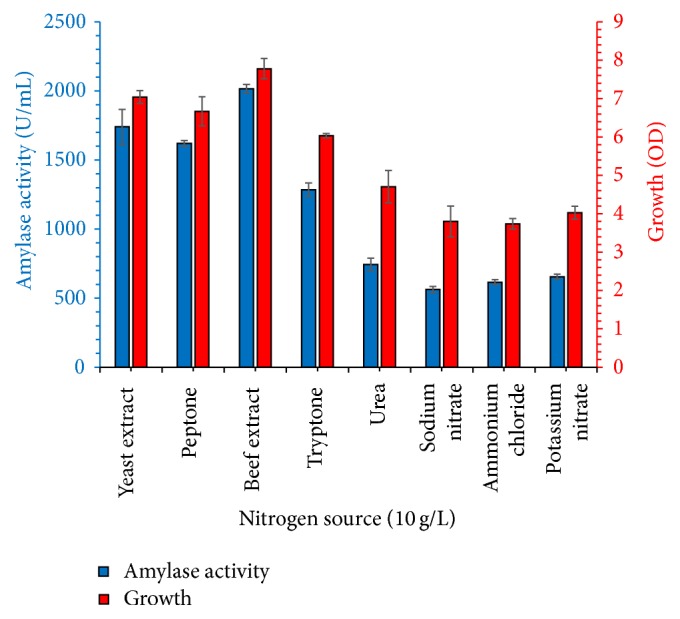
Effect of nitrogen source (10 g/L initial concentration) on biosynthesis of amylase from new thermophilic* Bacillus* sp. BCC 01-50. Experiment was performed in a molasses (30 g/L) mineral medium, and culture was incubated at 37°C for 60 h. The experiments were performed in triplicate and data presented in figure is average of three parallel experiments. Error bars are shown for standard deviation.

**Figure 6:**
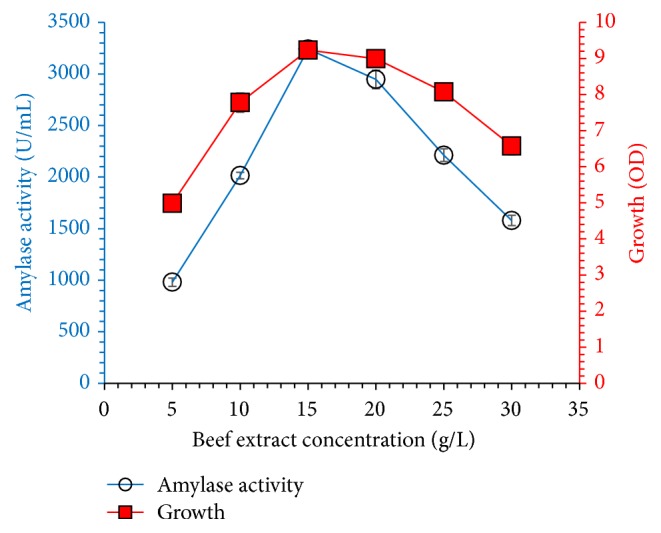
Effect of beef extract concentration (5–30 g/L) on amylase titer and cell growth. Experiment was performed in fermentation medium containing varying concentration of beef extract, and culture was incubated at 37°C for 60 h. The experiments were performed in triplicate and data presented in figure is average of three parallel experiments. Error bars are shown for standard deviation.

Various organic and inorganic compounds, potassium nitrate, ammonium chloride, sodium nitrate, urea, yeast extract, tryptone, beef extract, and peptone, were tested as a nitrogen source. Results shown in Figure 5 reveal that addition of 10 g/L beef extract enhanced the growth of* Bacillus* sp. and amylase yield. All tested organic nitrogen sources supported bacterial growth, but higher amylase titer was obtained when organic nitrogen sources were used as compared to inorganic nitrogen sources. All tested nitrogen sources supported bacterial growth, but higher amylase titer was obtained when organic nitrogen sources were used as compared to inorganic nitrogen sources. Our results are supported and in agreement with Qureshi et al. [14] for amylase production from organic nitrogen source. Fungal strain produced maximum amylase titer when grown in mineral medium containing ammonium sulfate as nitrogen source [22]. In another experiment, effect of beef extract concentration (5–30 g/L) on bacterial growth and amylase production was checked. Amylase production and bacterial growth were noted highest when 15 g/L beef extract was added in the medium and increase of concentration decreased the production and growth of the organism; results are shown in Figure 6.

**Figure 7:**
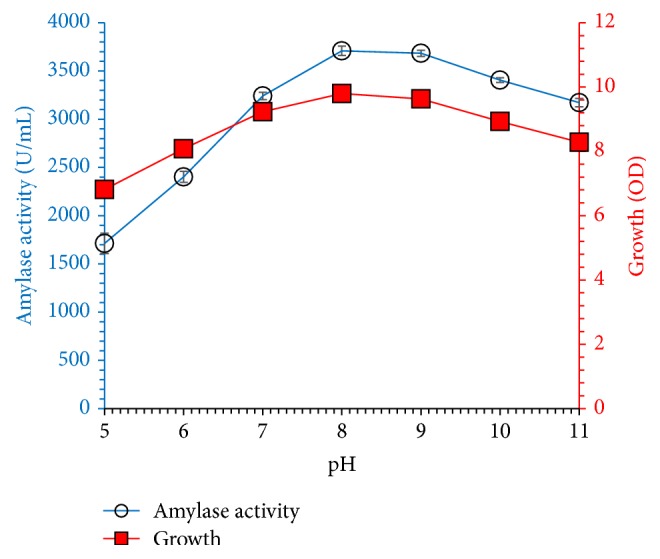
Effect of initial pH on amylase production and bacterial growth at 37°C for 60 h in a mineral medium containing molasses (30 g/L) and beef extract (15 g/L) as carbon and nitrogen sources, respectively. The experiments were performed in triplicate and data presented in figure is average of three parallel experiments. Error bars are shown for standard deviation.

**Figure 8:**
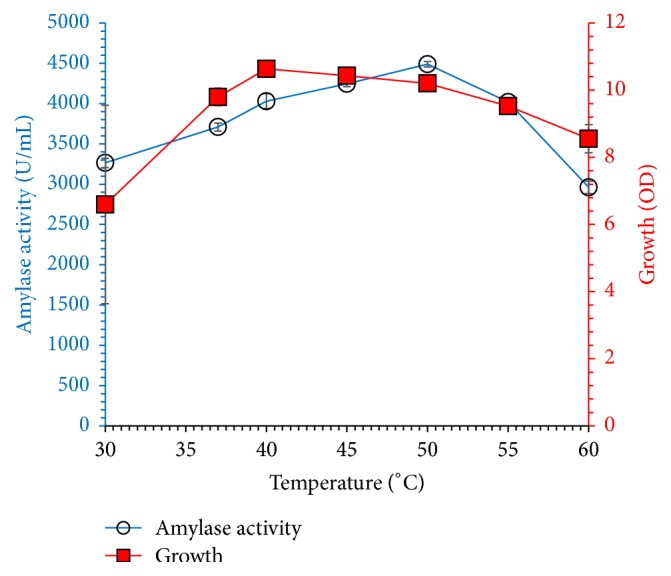
Effect of fermentation temperature on amylase production and microbial growth for 60 h. The medium initially contained molasses (30 g/L) and beef extract (15 g/L) as carbon and nitrogen sources, respectively. The initial pH was 8. The experiments were performed in triplicate and data presented in figure is average of three parallel experiments. Error bars are shown for standard deviation.

Figure 7 demonstrates the influence of initial pH (5.0 to 11.0) on amylase synthesis by* Bacillus* sp. in minimal medium containing 30 g/L molasses and 15 g/L beef extract incubated at 37°C for 60 h in shaking conditions (150 rpm). Microbial growth and amylase production were noted highest at pH 8.0, which indicates alkaliphilic nature of strain. Moreover, no significant reduction was observed in microbial growth and amylase titer in alkaline pH. Microbial growth and amylase production were noted highest at pH 8.0, which indicates alkaliphilic nature of strain. Literature supports optimum alkaline pH for amylase production from* Bacillus cereus* under solid state fermentation [23]. In contrast to our results, [22] have obtained maximum amylase concentration in acidic pH (6.0) by fungal strains isolated and screened from legume seeds. Generally, alkaliphilic microbial strains and their products are of commercial importance and especially alkaline stable amylase could be applied in detergents industry.  Figure 8 shows the effect of fermentation temperature on the synthesis of amylase by* Bacillus* sp. (30–60°C) grown in fermentation medium containing 30 g/L molasses and 15 g/L beef extract pH 8.0 which was incubated for 60 h. Enzyme concentration increased with incubation temperature and maximum amylase yield was noted at 50°C; further increase of temperature reduced amylase titer due to decreased microbial growth at elevated temperature. Enzyme concentration increased with incubation temperature and maximum amylase yield was noted at 50°C; further increase of temperature reduced amylase titer due to decreased microbial growth at elevated temperature. Similarly, Vijayaraghavan et al. and Asad et al. [3, 23] obtained maximum amylase secretion from* Bacillus cereus* IND4 and* Bacillus* sp. WA21 at 45°C on starch agar medium, whereas Abdullah et al. [2] reported maximum amylase production at moderate temperature (30°C) from the fungal strain. Thermophilic microorganisms are industrially very important due to the production of thermophilic enzymes and these strains also reduce the chances of contamination during fermentation. Our study suggested that amylase production from* Bacillus* sp. thermophilic strain is growth-dependent.

**Figure 9:**
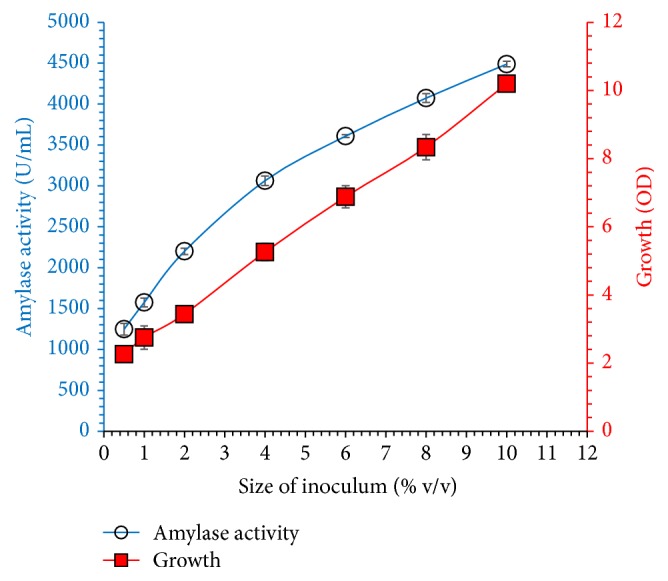
Size of inoculum (0.5 to 10% v/v) on amylase activity and bacterial growth was studied. Bacterial culture was grown for 24 h in seeds culture and different concentration was added in the fermentation medium. Culture incubated at 50°C for 60 h, and initial pH was adjusted to 8 in shaking incubator. The experiments were performed in triplicate and data presented in figure is average of three parallel experiments. Error bars are shown for standard deviation.

Figure 9 shows the effect of inoculum size (0.5 to 10% v/v) on cell growth and extracellular amylase secretion. Highest amylase concentration was achieved when 10 (% v/v) of 24 h old seeds culture was inoculated into the fermentation medium. This study suggested that amylase production from* Bacillus* sp. thermophilic strain is growth-dependent. Our results are in line with literature for enzyme production from various strains in terms of inoculum size. The size of inoculum and age of inoculum play a significant role in the rate of fermentation. Therefore, optimization of inoculum age should always be considered as important fermentation parameter.

### 3.2. Characterization of Crude Enzyme

**Figure 10:**
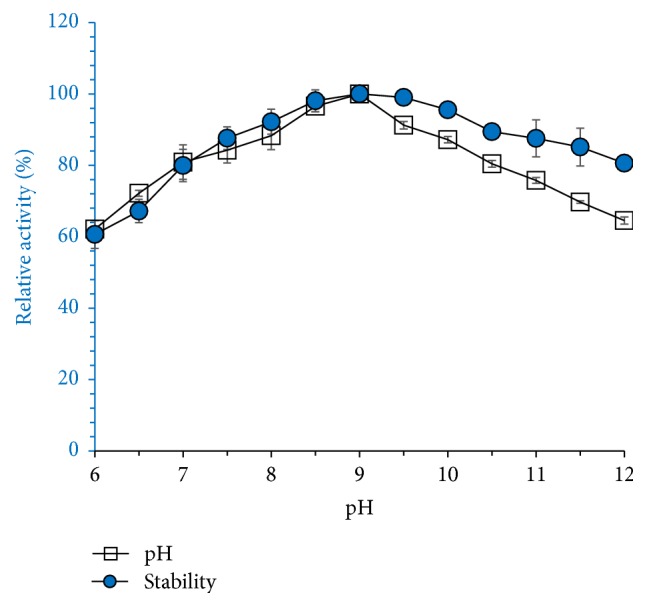
Effect of pH on amylase activity and stability. For evaluating pH activity, percentage of relative activity was measured by incubating enzyme with substrate at different pH values (512). Buffers used are as follows: sodium acetate buffer, pH 5; phosphate buffer, pH 6.0–8; glycine–NaOH buffer, pH 9.0–12.0. pH stability of crude amylase was determined by measuring amylase activity by DNS method. Crude preparation was incubated for 1 h, at specified pH values (above-mentioned buffers) at 35°C, prior to addition of substrate, and % of relative activity was determined. Results are the average of triplicate experiment.

**Figure 11:**
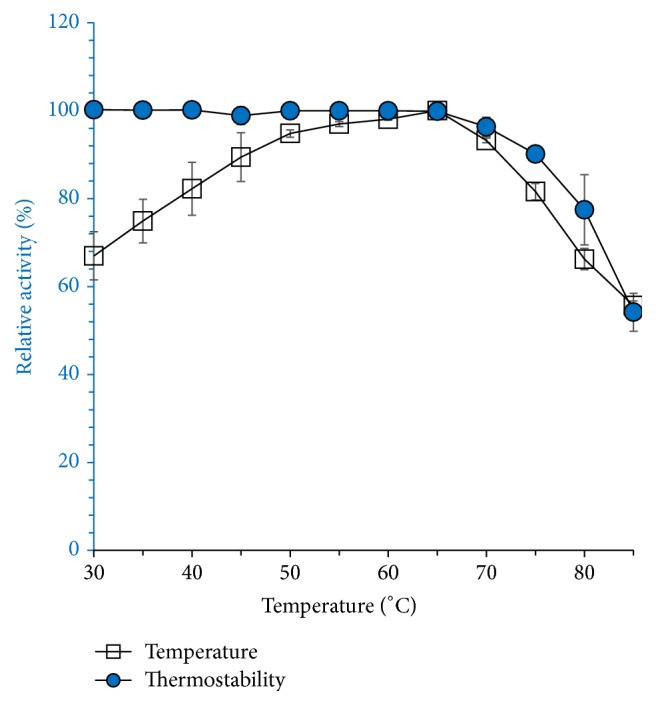
Effect of temperature on amylase activity. Enzyme was mixed with substrate and incubated at different temperature for 1 h. % of relative activity was measured under assay conditions. Thermostability of amylase was determined by incubating enzyme at different temperature without addition of substrate for 1 h. Percentage of relative activity was checked under assay conditions. Results are the average of triplicate experiment.

Crude amylase was characterized in terms of pH, temperature, pH stability, and thermostability.  Figure 10 shows the effect of pH and pH stability for enzyme-substrate catalysis reaction. Amylase activity was found in the pH range of 8–10, with maximal activity at pH 9. Enzyme activity inclined with pH towards alkaline range that indicates alkaliphilic nature of the enzyme. Crude amylase was stable in alkaline pH 8–10 for 1 h (Figure 9). Enzyme stability was comparatively low in acidic and neutral pH ranges (5.0–7.0). Crude amylase was characterized in terms of pH, temperature, pH stability, and thermostability. Enzyme stability was comparatively low in acidic and neutral pH ranges (5.0–7.0). The results of this study clearly indicate the industrially important characteristic alkaliphilic nature of the enzyme and it makes suitable additive. pH stability of crude amylase could be compared with* Bacillus methylotrophicus* strain P11-2 [24]. The* B. methylotrophicus* amylase was stable for 1 h at pH 6.0–9.0, with the reduction in the residual activities at pH 4.0 and 5.0. Amylase was thermostable with optimum temperature for activity at 65°C. The results are comparable with an amylase from* Nocardiopsis* sp. where the similar profile is reported by Stamford et al. [25]. The results of this study clearly indicate the industrially important characteristic alkaliphilic nature of the enzyme and it makes suitable additive. Crude amylase of* Bacillus* sp. BCC 01-50 was active in the temperature range of 30–95°C, the optimum being at 65°C; results are shown in Figure 11. Crude amylase was highly stable in the temperature range 40–70°C, with maximum stability at 70°C. However, the enzymatic stability decreased above 70°C. Our results indicate high thermal and alkali-tolerant nature of the enzyme, a dual extreme characteristic of the enzyme, because the enzyme is 100% stable up to 65°C and 9.0 pH.

### 3.3. Applications of Crude Amylase

**Figure 12:**
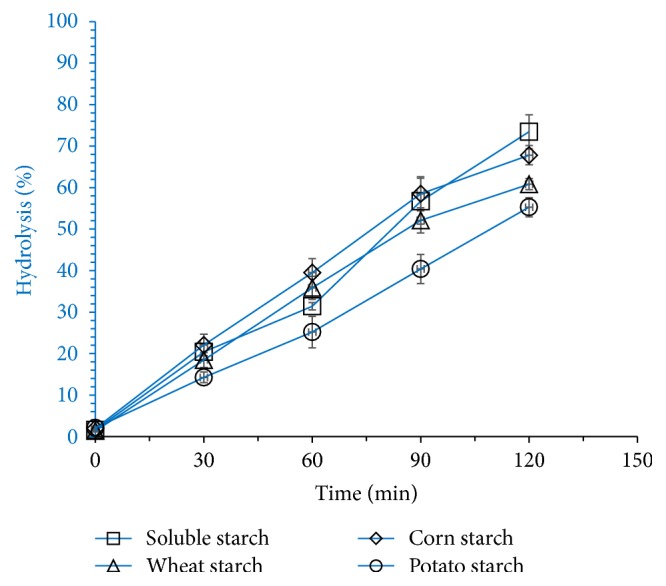
Hydrolysis efficiency of amylase produced by* Bacillus* sp. BCC 01-50 towards different starchy materials including soluble starch, wheat starch, corn starch, and potato starch. 1% starch was mixed with crude amylase enzyme and reaction was incubated at 65°C for 120 minutes. Samples were collected at different interval and hydrolysis efficiency was calculated according to given equation in Materials and Methods.

The starch stained cotton fabric pieces were washed with water, detergents, crude enzyme, and detergent plus enzyme. The efficiency was determined from washout on the basis of the removal of starch content and efficiency can be expressed as the higher the starch content, the higher the efficiency. The washing efficiency was very low in tap water but improved with the addition of detergent and enzyme alone plus detergent. In our study, washing efficiency was significantly improved, when detergent plus crude enzyme was used. All the results of washing efficiency have been proved for the efficient use of *α*-amylase in washing process and are summarized in Tables 2 and 3. The efficiency of crude *α*-amylase produced from* Bacillus* sp. BCC 01-50 towards different raw starch sources including wheat, potato and corn starches, and soluble starch was investigated by monitoring the extent of hydrolysis of various starch material in a short duration of time; results are depicted in Figure 12. The rate of hydrolysis of soluble starch, wheat, potato, and corn starches at 1% concentration was 73.43, 60.81, 55.26, and 67.81% in a period of 120 min, respectively. The starch stained cotton fabric pieces were washed with water, detergents, crude enzyme, and detergent + enzyme. Washing efficiency greatly improved with crude amylase. Results are in agreement with the studies of Dhingra et al., Prakash and Jaiswal, and Shukla and Singh [17, 26, 27]. The results are very promising and washing efficiency of detergent improved when crude amylase was added. The efficiency of crude *α*-amylase produced from* Bacillus* sp. BCC 01-50 towards different raw starch sources including wheat, potato and corn starches, and soluble starch was investigated and compared with previous studies. Full comparison with other amylase producing bacterial species is not possible due to the different reaction conditions, different strains, and fermentation conditions [17, 28]. Yang and Liu, 2004, and Shukla and Singh, 2015, applied purified amylase with 1% raw starch material and incubation time was 4 h, while Yang and Liu [28] also applied purified enzyme to hydrolyze various raw starch materials at concentration of 5% and time required for hydrolysis was 24 h. Mitsuiki et al. [8] evaluated starch hydrolysis of potato and corn raw starches with amylase enzyme produced by different* Bacillus* species. 50 mg of starch was mixed with 2 mL of amylase enzyme and reaction mixture.

## 4. Conclusion

Increasing amylase demand, high nutrient cost, and environmental pollution have compelled utilizing agroindustrial residues as inexpensive nutrients for enzyme production, so the present study has been taken up with a view to explore the possible use of agroindustrial residues as nutrient source for the production of amylase through submerged fermentation conditions because the use of inexpensive carbon and nitrogen sources can reduce the cost of production. In the present study, cultural conditions were optimized for amylase production from* Bacillus* sp. BCC 01-50 using molasses in shake flasks as a batch culture technique. Highest amylase titer of ~4500 U/mL was obtained when 30 g/L of molasses was used. Our study suggests that molasses could be potentially very cost-effective energy source for commercial amylase production and that utilization of molasses not only reduces the cost of fermentation medium but also helps to reduce pollution problem due to agroindustrial residues. Moreover, crude amylase applicability showed great potential for its application in detergent industry and saccharification of starchy materials. Results obtained herein are more promising for the inexpensive production of amylase by using agroindustrial residue as cost-effective carbon source for biotechnological applications.

## Figures and Tables

**Table 1:** Growth kinetics of *Bacillus* sp. BCC 01-50 and sugar consumption in synthetic medium.

Time h	Total sugar g/L	Dry cell weight g/L	^a^ *r*_*x*_ (g/L/h)	^b^ *r*_*s*_ (g/L/h)	^c^ *Y*_*x*/*s*_
0	17.8	0.20	—	—	—
12	16.1	0.65	0.45	1.76	0.25
24	14.1	1.06	0.85	3.70	0.23
36	11.1	1.88	1.67	6.70	0.24
48	5.75	2.25	2.04	12.11	0.16
60	2.74	2.50	2.29	15.12	0.15
72	1.77	2.06	1.85	16.09	0.11
84	0.95	1.33	1.12	16.91	0.06
96	0.56	1.11	0.91	17.30	0.05
108	0.46	0.90	0.70	17.40	0.04
120	0.32	0.65	0.44	17.54	0.02

^a^ *r*_*x*_ biomass formation rate, *dx*/*dt* (g/L/h); ^b^*r*_*s*_ sugar consumption rate, *ds*/*dt* (g/L/h); ^c^*Y*_*x*/*s*_ biomass yield from sugar, *r*_*x*_/*r*_*s*_ (g/g). Results shown in above table are average of triplicate parallel experiments.

**Table 2:** Washing performance with water and crude amylase produced from *Bacillus* sp. BCC 01-50.

Washing agent	% of starch contents in washout
Tap water as control	11.23
Crude amylase	43.28

**Table 3:** Washing performance of commercial detergents and with crude amylase produced from *Bacillus* sp. BCC 01-50.

Detergent 5 g/L	Only detergent	Detergent + amylase
Ariel	28.29	71.23
Omo	33.97	75.39
Bright	35.10	79.33
Surf Excel	31.67	71.45
Bonus	32.91	73.54
Rin	33.63	69.73
